# Efficacy, Safety, Tolerability, and Pharmacokinetics of MMV390048 in Acute Uncomplicated Malaria

**DOI:** 10.4269/ajtmh.22-0567

**Published:** 2022-12-05

**Authors:** Rezika Mohammed, Mezgebu Silamsaw Asres, Esayas Kebede Gudina, Wondimagegn Adissu, Hilary Johnstone, Anne Claire Marrast, Cristina Donini, Stephan Duparc, Daniel Yilma

**Affiliations:** ^1^ Department of Internal Medicine, University of Gondar Hospital, Gondar, Ethiopia;; ^2^ Jimma University Clinical Trial Unit, Jimma University Institute of Health, Jimma, Ethiopia;; ^3^ Department of Internal Medicine, Jimma University Institute of Health, Jimma, Ethiopia;; ^4^ School of Medical Laboratory Sciences, Jimma University Institute of Health, Jimma, Ethiopia;; ^5^ HJ-Clinical Trial Consultancy, George, South Africa;; ^6^ Medicines for Malaria Venture, Geneva, Switzerland

## Abstract

An open label, phase IIa study conducted in Ethiopia evaluated the efficacy, safety, tolerability, and pharmacokinetics of a single 120-mg dose of the phosphatidylinositol 4-kinase inhibitor MMV390048 in *Plasmodium vivax* malaria. The study was not completed for operational reasons and emerging teratotoxicity data. For the eight adult male patients enrolled, adequate clinical and parasitological response at day 14 (primary endpoint) was 100% (8/8). Asexual parasites and gametocytes were cleared in all patients by 66 and 78 hours postdose, respectively. There were two recurrent *P. vivax* infections (days 20 and 28) and a new *Plasmodium falciparum* infection (day 22). MMV390048 exposure in *P. vivax* patients was lower than previously observed for healthy volunteers. Mild adverse events, mainly headache and gastrointestinal symptoms, were reported by eight patients. Single-dose MMV390048 (120 mg) rapidly cleared asexual parasites and gametocytes in patients with *P. vivax* malaria and was well tolerated.

Malaria remains major threat to global health.^1^ The need for new measures to support malaria elimination and the emergence of drug-resistant *Plasmodium* strains requires the discovery and development of new antimalarial drugs.^2^

MMV390048 is a phosphatidylinositol 4-kinase inhibitor with in vitro activity against all *Plasmodium* life cycle stages, excepting late-stage hypnozoites in the liver.^3^^–^^5^ MMV390048 lacks cross-resistance with current antimalarial drugs,^6^ and in vivo studies showed both transmission blocking and chemoprotective activity.^6^ In phase I clinical studies, MMV390048 was well tolerated at doses up to 120 mg.^7^^,^^8^ Pharmacokinetic/pharmacodynamic modeling predicted that an adequate clinical and parasitological response (ACPR) > 80% would be achieved at day 14 posttreatment with a single 120-mg dose of MMV390048 with 92% certainty.^8^ Thus, MMV390048 has potential as a single-dose therapy for the treatment and control of malaria,^6^^–^^8^ although this would require combination with a suitable partner drug with antimalarial activity.

This open label, adaptive, phase IIa study was designed to evaluate the efficacy, safety, tolerability, and pharmacokinetics of a single 120-mg dose of MMV390048 in adult patients with uncomplicated malaria. The study was conducted between October 6, 2017 and January 5, 2018 at two hospitals in Ethiopia (in Gondar and Jimma). Recruitment was suspended on December 4, 2017 to allow assessment of a teratogenicity signal in a concurrent investigation in rodents.^9^ Although approval to restart the study was obtained in August 2019, the study was terminated on October 21, 2020 for operational reasons related to the coronavirus 2019 pandemic and because of the teratogenic findings. Herein, we briefly describe the study design and report the abbreviated dataset on the eight enrolled patients.

The study protocol was approved by the independent Ethics Committees of the College of Public Health and Medical Sciences, Jimma University (now the Institute of Health Institutional Review Board), the Institutional Review Board of the University of Gondar, Ethiopia, and the Ethiopian National Research Ethics Review Committee and Ethiopian Food and Drug Administration, Addis Ababa, and was registered with ClinicalTrials.gov (NCT02880241). Study conduct conformed to the national regulatory requirements of Ethiopia and the Declaration of Helsinki.

Planned enrollment was for three *P. vivax* and three *P. falciparum* cohorts of 17 patients each (102 patients in total). MMV390048 was supplied as 20-mg tablets (Medicines for Malaria Venture, Geneva, Switzerland). The first *P. vivax* and *P. falciparum* cohorts were to receive a single 120-mg oral dose of MMV390048 in the fasted state, with de-escalating dosing of subsequent cohorts determined by the results obtained. Four patients were to be enrolled into the *P. vivax* arm and followed until day 14 before enrolment into the *P. falciparum* arm was to begin, dependent on an acceptable review by the safety review team.

Eligible patients were adults aged 18–55 years, weighing 40–90 kg with microscopically confirmed *P. vivax* or *P. falciparum* monoinfection (1,000–40,000 asexual parasites per microliter of blood), fever or history of fever within the previous 48 hours for *P. vivax* and 24 hours for *P. falciparum*, and no signs or symptoms of severe or complicated malaria. Patients were admitted and received a single oral dose of 120-mg MMV390048 on day 0 and remained as inpatients until day 3 and two consecutive negative parasite assessments. Outpatient follow-up visits were made on days 7, 10/11, 14, 17/18, 21, 24/25, and 28. Rescue therapy for *P. vivax* was chloroquine. At the time of the study start, primaquine was not the standard of care in Ethiopia for patients living in malaria endemic regions. However, primaquine radical cure was administered to six of eight patients at the investigator’s discretion after a negative glucose-6-phosphate dehydrogenase test.

Giemsa-stained thick and thin blood films for parasite identification and enumeration were prepared using standard methods.^10^ Species-specific *Pf_* and *Pv*_18S rRNA quantitative polymerase chain reaction (qPCR) was consistent with published protocols.^11^ MMV390048 plasma concentrations were determined using a validated assay and analyzed using non-compartmental methods.^7^^,^^8^ Adverse events were coded according to MedDRA version 23.0.

The primary outcome for *P. vivax* malaria was unadjusted ACPR at day 14, defined as complete clearance of microscopically detected parasitemia without previous treatment failure. This enabled a rapid readout of clinical efficacy (i.e., early treatment failure) and safety for dose adjustment of subsequent cohorts and progression to enrolment of the *P. falciparum* arm. Secondary efficacy outcomes for *P. vivax* were ACPR and recurrence rate at day 28. Safety outcomes included the frequency of adverse events up to day 35, and signs and symptoms of malaria up to day 28. Parasite clearance kinetics and MMV390048 pharmacokinetics were also planned analyses. Summary statistics were prepared for efficacy and safety data; no inferential statistical analysis was performed for this abbreviated dataset.

The eight enrolled patients were males, self-defined as black, mean age 24.5 years (range 20–50 years), with a mean (SD) body mass index of 18.1 (1.2) kg/m^2^. All were infected with *P. vivax* malaria with a mean (SD) pre-dose asexual parasite count of 6,406 (6767) parasites/μL.

The primary endpoint of ACPR at day 14 was 100% (8/8). Asexual parasites were cleared by 24 hours postdose in four patients, by 48 hours in two patients, and by 66 hours in the remaining two patients (Figure 1). All patients remained parasite free until day 14. Recurrent *P. vivax* infection was reported in two patients (days 20 and day 28), both of whom received primaquine. A new *P. falciparum* infection was detected on day 22 in one patient (no primaquine). Thus, ACPR at day 28 was 62.8% (5/8) in the modified intention-to-treat analysis, and 71.4% (5/7) when the patient with the new *P. falciparum* infection was excluded.

**Figure 1.**
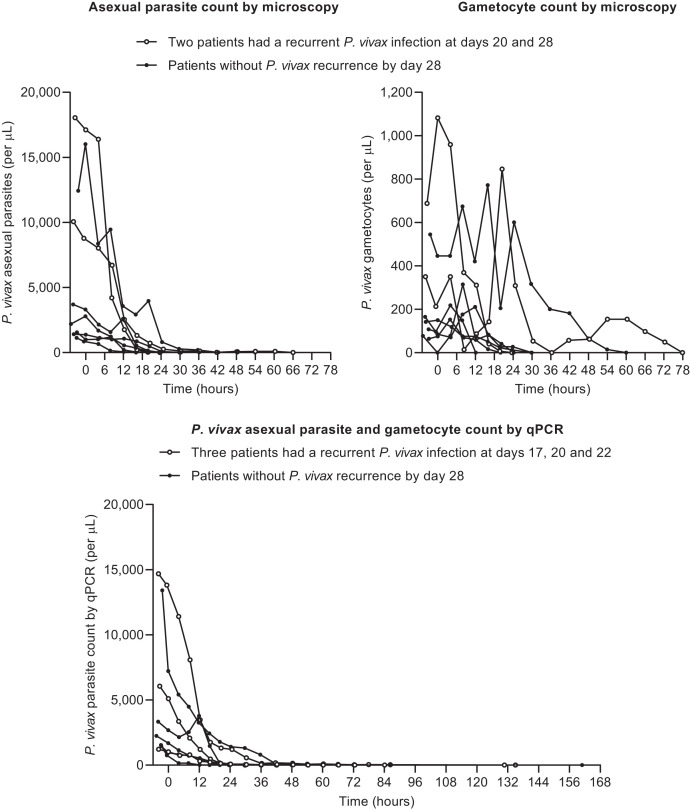
Parasite counts for *P. vivax* asexual forms and gametocytes evaluated using light microscopy and qPCR from pre-dose until parasite clearance. MMV390048 was dosed at time 0.

Gametocytes were detected in all patients at baseline and were cleared by 24 hours postdose in four patients, by 30 hours in two patients, and by 78 hours in two patients (Figure 1). Gametocytes were detectable in the two patients with *P. vivax* recurrence on days 20 and 28.

Using qPCR, parasite clearance was achieved between 20 and 161 hours (Figure 1), with three recurrences detected (days 17, 20, and 22). Overall, MMV390048 rapidly cleared both asexual parasites and gametocytes, although recurrent infections before day 28 suggest that greater drug exposure is needed to maintain efficacy, particularly in patients with high baseline parasitemia (Figure 1).

MMV390048 pharmacokinetic parameters are shown in Table 1. Although *C*_max_ (peak plasma concentration) was comparable with studies in healthy volunteers, *t*_1/2_ (estimated elimination phase half-life) and AUC_inf_ (area under the concentration–time curve from 0 hour to infinity) were lower than previously reported (Table 1).^7^^,^^8^ The reasons for these differences are currently unknown, but drug exposures between patients were highly variable, most likely because of variable bioavailability and the small number of patients available for comparison across the studies. Less likely, but observed for some other antimalarials, are the possible effects on drug metabolism of inflammation caused by *P. vivax* infection^12^^–^^14^ and pharmacogenetic differences.^15^^,^^16^

**Table 1:** Plasma pharmacokinetic parameters for a single 120-mg dose of MMV390048 in adult patients with *P. vivax* malaria from Ethiopia compared with previously reported data from healthy volunteers from Australia^8^ and South Africa^7^

Pharmacokinetic parameter	Ethiopia (*N* = 8)	Australia (*N* = 6)	South Africa (*N* = 5)
*C*_max_, μg/mL	0.5 (59.0)	1.1 (36.6)	0.5 (85.5)
*T*_max_, hours	2.0 (2.0–12.0)	1.0 (1.0–3.0)	1.0 (1.0–3.0)
*t*_1/2_, hours	37.4 (57.7)	2135.2 (143.1–271.5)*	206.1 (32.9)
AUC_last_, μg⋅h/mL	16.7 (57.7)	123.2 (28.5)	Not available
AUC_inf_, μg⋅h/mL	17.3 (57.0)	137.8 (33.6)	82.6 (165.8)

Values are geometric mean (coefficient of variation), except for *T*_max_, which is median (range). AUC_inf_ = area under the concentration–time curve from 0 hour to infinity; AUC_last_ = area under the concentration–time curve from 0 hour to the last measured time point;* C*_max_ = peak plasma concentration; *t*_1/2_ = estimated elimination phase half-life; *T*_max_ = time point at which peak plasma concentration is reached.

* The reported *t*_1/2_ in this study was median (range).

There were no deaths, serious adverse events, or adverse events leading to study discontinuation. A total of 27 adverse events were reported during the study across all eight patients (Table 2). All adverse events were grade 1 (mild); headache and abdominal discomfort were most commonly reported (Table 2). Seven drug-related adverse events were reported in four patients: abdominal discomfort, abdominal pain, constipation, dyspepsia, headache, neutropenia, and decreased hemoglobin.

**Table 2:** Adverse events of any cause

Adverse event	MMV390048 (*N* = 8) *n* (%) patients/*n* events
At least one adverse event	8 (100.0)/27
Headache	5 (62.5)/6
Abdominal discomfort	2 (25.0)/2
Abdominal pain	1 (12.5)/1
Constipation	1 (12.5)/1
Dyspepsia	1 (12.5)/1
Nausea	1 (12.5)/1
Oral discomfort	1 (12.5)/1
Vomiting	1 (12.5)/1
Ascariasis	1 (12.5)/1
Carbuncle	1 (12.5)/1
Hookworm infection	1 (12.5)/1
*Plasmodium falciparum* infection	1 (12.5)/1
Upper respiratory tract infection	1 (12.5)/1
Decreased appetite	1 (12.5)/2
Neutropenia*	1 (12.5)/1
Sinus tachycardia*	1 (12.5)/1
Fatigue	1 (12.5)/1
Hemoglobin decreased*	1 (12.5)/1
Arthralgia	1 (12.5)/1
Urinary tract discomfort	1 (12.5)/1

* Adverse event of special interest.

There were three adverse events of special interest occurring in two patients. One patient had neutropenia, considered possibly drug related. This event started on day 2 (baseline neutrophil count 3.39 × 10^9^/L; 0.97 × 10^9^/L on day 2) and spontaneously resolved by day 6 and was concurrent with an upper respiratory tract infection that started on day 3. One patient had sinus tachycardia on day 1 that resolved the same day and was considered related to malaria infection. This patient also had decreased hemoglobin on day 2 (baseline 13.9 g/dL; 11.8 g/dL on day 2) that had resolved by day 6 and was considered related to drug treatment and malaria infection.

Clinical laboratory tests showed no drug-related trends. Baseline low platelet and hemoglobin levels, consistent with malaria infection, tended to improve throughout the study. There were no other safety concerns.

In summary, a single oral dose of 120-mg MMV390048 rapidly cleared asexual parasites and gametocytes in eight male patients with *P. vivax* malaria. ACPR was 100% at day 14, but with recurrent *P. vivax* infection in two patients (days 20 and 28). MMV390048 drug exposures were lower than expected based on previous findings in healthy volunteers. There were no safety or tolerability concerns with MMV390048 administration.

## References

[1] World Health Organization , 2021. World malaria report. Available at: https://www.who.int/teams/global-malaria-programme/reports/world-malaria-report-2021. Accessed July 11, 2022.

[2] Duffey M Blasco B Burrows JN Wells TNC Fidock DA Leroy D , 2021. Assessing risks of *Plasmodium falciparum* resistance to select next-generation antimalarials. Trends Parasitol 37: 709–721. 34001441 10.1016/j.pt.2021.04.006 PMC8282644

[3] Mustiere R Vanelle P Primas N , 2020. Plasmodial kinase inhibitors targeting malaria: recent developments. Molecules 25: 5949. 33334080 10.3390/molecules25245949 PMC7765515

[4] Sternberg AR Roepe PD , 2020. Heterologous expression, purification, and functional analysis of the *Plasmodium falciparum* phosphatidylinositol 4-kinase iiibeta. Biochemistry 59: 2494–2506. 32543181 10.1021/acs.biochem.0c00259

[5] Ghidelli-Disse S 2014. Identification of *Plasmodium* PI4 kinase as target of MMV390048 by chemoproteomics. Malar J 13 *(* Suppl 1 *):* 38.24479524

[6] Paquet T 2017. Antimalarial efficacy of MMV390048, an inhibitor of *Plasmodium* phosphatidylinositol 4-kinase. Sci Transl Med 9: eaad9735. 28446690 10.1126/scitranslmed.aad9735 PMC5731459

[7] Sinxadi P 2020. Safety, tolerability, pharmacokinetics, and antimalarial activity of the novel *Plasmodium* phosphatidylinositol 4-kinase inhibitor MMV390048 in healthy volunteers. Antimicrob Agents Chemother 64: e01896-19. 31932368 10.1128/AAC.01896-19 PMC7179259

[8] McCarthy JS 2020. A phase 1, placebo-controlled, randomized, single ascending dose study and a volunteer infection study to characterize the safety, pharmacokinetics, and antimalarial activity of the *Plasmodium* phosphatidylinositol 4-kinase inhibitor MMV390048. Clin Infect Dis 71: e657–e664. 32239164 10.1093/cid/ciaa368 PMC7744986

[9] European Medicines Agency , 2017. Guideline on clinical development of fixed combination medicinal products. Available at: https://www.ema.europa.eu/en/documents/scientific-guideline/guideline-clinical-development-fixed-combination-medicinal-products-revision-2_en.pdf. Accessed June 2, 2022.

[10] World Health Organization , 2016. Malaria microscopy quality assurance manual, version 2. Available at: https://apps.who.int/iris/handle/10665/204266. Accessed November 14, 2021.

[11] Gruenberg M 2020. Utility of ultra-sensitive qPCR to detect *Plasmodium falciparum* and *Plasmodium vivax* infections under different transmission intensities. Malar J 19: 319. 32883308 10.1186/s12936-020-03374-7 PMC7469345

[12] Mimche SM Lee CM Liu KH Mimche PN Harvey RD Murphy TJ Nyagode BA Jones DP Lamb TJ Morgan ET , 2019. A non-lethal malarial infection results in reduced drug metabolizing enzyme expression and drug clearance in mice. Malar J 18: 234. 31299982 10.1186/s12936-019-2860-5 PMC6624958

[13] De-Oliveira AC Carvalho RS Paixao FH Tavares HS Gueiros LS Siqueira CM Paumgartten FJ , 2010. Up- and down-modulation of liver cytochrome P450 activities and associated events in two murine malaria models. Malar J 9: 81. 20307316 10.1186/1475-2875-9-81 PMC2858213

[14] McCarthy JS Furner RL Van Dyke K Stitzel RE , 1970. Effects of malarial infection on host microsomal drug-metabolizing enzymes. Biochem Pharmacol 19: 1341–1349. 5513924 10.1016/0006-2952(70)90049-3

[15] Desta Z Zhao X Shin JG Flockhart DA , 2002. Clinical significance of the cytochrome P450 2C19 genetic polymorphism. Clin Pharmacokinet 41: 913–958. 12222994 10.2165/00003088-200241120-00002

[16] Kalow W , 1997. Pharmacogenetics in biological perspective. Pharmacol Rev 49: 369–379. 9443163

